# Three cases of chronic photodermatitis successfully treated with upadacitinib

**DOI:** 10.1093/skinhd/vzaf049

**Published:** 2025-07-23

**Authors:** Hissa Al-Marri, Wadha Al-Shafi, Mohammed Al-Abdula, Mohammed Al-Jaber, Ayda AlHammadi, Aysha Al-Malki, Hanof Ahmed, Maryam Al-Jaidah, Fatima Al-Khawaja, Seena Manjooran, Febu Joy, Fareed Ahmad, Joerg Buddenkotte, Martin Steinhoff

**Affiliations:** Department of Dermatology and Venereology, Hamad Medical Corporation, Doha, Qatar; Dermatology Institute, Academic Health System, Hamad Medical Corporation, Doha, Qatar; Department of Dermatology and Venereology, Hamad Medical Corporation, Doha, Qatar; Dermatology Institute, Academic Health System, Hamad Medical Corporation, Doha, Qatar; Department of Dermatology and Venereology, Hamad Medical Corporation, Doha, Qatar; Dermatology Institute, Academic Health System, Hamad Medical Corporation, Doha, Qatar; Department of Dermatology and Venereology, Hamad Medical Corporation, Doha, Qatar; Dermatology Institute, Academic Health System, Hamad Medical Corporation, Doha, Qatar; Department of Dermatology and Venereology, Hamad Medical Corporation, Doha, Qatar; Dermatology Institute, Academic Health System, Hamad Medical Corporation, Doha, Qatar; Department of Dermatology and Venereology, Hamad Medical Corporation, Doha, Qatar; Dermatology Institute, Academic Health System, Hamad Medical Corporation, Doha, Qatar; Department of Dermatology and Venereology, Hamad Medical Corporation, Doha, Qatar; Dermatology Institute, Academic Health System, Hamad Medical Corporation, Doha, Qatar; Department of Dermatology and Venereology, Hamad Medical Corporation, Doha, Qatar; Dermatology Institute, Academic Health System, Hamad Medical Corporation, Doha, Qatar; Department of Dermatology and Venereology, Hamad Medical Corporation, Doha, Qatar; Dermatology Institute, Academic Health System, Hamad Medical Corporation, Doha, Qatar; Department of Dermatology and Venereology, Hamad Medical Corporation, Doha, Qatar; Dermatology Institute, Academic Health System, Hamad Medical Corporation, Doha, Qatar; Department of Dermatology and Venereology, Hamad Medical Corporation, Doha, Qatar; Dermatology Institute, Academic Health System, Hamad Medical Corporation, Doha, Qatar; Department of Dermatology and Venereology, Hamad Medical Corporation, Doha, Qatar; Dermatology Institute, Academic Health System, Hamad Medical Corporation, Doha, Qatar; Translational Research Institute, Academic Health System, Hamad Medical Corporation, Doha, Qatar; Department of Dermatology and Venereology, Hamad Medical Corporation, Doha, Qatar; Dermatology Institute, Academic Health System, Hamad Medical Corporation, Doha, Qatar; Translational Research Institute, Academic Health System, Hamad Medical Corporation, Doha, Qatar; Department of Dermatology and Venereology, Hamad Medical Corporation, Doha, Qatar; Dermatology Institute, Academic Health System, Hamad Medical Corporation, Doha, Qatar; Translational Research Institute, Academic Health System, Hamad Medical Corporation, Doha, Qatar; Department of Medicine, Weill Cornell Medicine-Qatar, Doha, Qatar; College of Medicine, Qatar University, Doha, Qatar; College of Health and Life Sciences, Hamad Bin Khalifa University, Doha, Qatar; Department of Dermatology, Weill Cornell Medicine, New York, NY, USA

## Abstract

Photodermatoses represent a varied collection of skin disorders characterized by cutaneous reactions provoked by exposure to ultraviolet radiation. The disease mechanism is based on an immune-mediated delayed hypersensitivity reaction. Clinical presentation of patients with photodermatosis includes pigmentary changes, erythema and/or lichenification, with interindividual differences in lesion morphology and distribution. Most patients report associated symptoms of burning sensation and itchiness as their chief complaint, severely affecting their quality of life. Management of photodermatoses involves both preventive measures and medical management applying topical steroids and calcineurin inhibitors, or systemic immunosuppressives, with variable degree of success and adverse events. Here, we report three cases of chronic photodermatitis treated with upadacitinib, a selective Janus kinase 1 inhibitor, as monotherapy, which resulted in fast, significant and sustainable improvement of signs and symptoms, with a favourable safety and tolerability profile.

## Teaser

Photodermatoses/actinic lichen planus (ALP) are abnormal delayed hypersensitivity (irritant, allergic) reactions to the ultraviolet light. Treatment of ALP is often recalcitrant and challenging. Here we report three cases of ALP successfully treated with upadacitinib, a selective oral JAK1 inhibitor.

**What is already known about this topic?**

- Photodermatoses are abnormal delayed hypersensitivity reactions to the ultraviolet component of sunlight.
- Treatment of actinic lichen planus (ALP) is often recalcitrant and challenging.
- Topical steroids, calcineurin inhibitors, systemic immunosuppressive medications and phototherapy have exhibited varying success.

**What does this topic add?**

- We report the first three cases of ALP successfully treated with upadacitinib.
- The Janus kinase/signal transducer and activator of transcription pathway appears to be a valid drug target for the treatment of ALP.

Photodermatoses are abnormal delayed hypersensitivity (irritant, allergic) reactions to the ultraviolet (UV) component of sunlight. Photodermatoses can be idiopathic [e.g. polymorphic light eruption (PLE) or chronic actinic dermatitis (CAD)], with PLE constituting the most common type of acquired chronic photodermatosis (CPD) (10–20% prevalence).^1^ CAD is mainly observed in men older than 50 years of age. Most patients present in the acute phase of erythema, often accompanied by papules, oedematous skin, superficial pityriasiform scaling on sun-exposed areas, burning pain and pruritus. In the chronic stage, patients predominantly present with lichenified and hyperpigmented skin. The morphology, distribution and pigmentation vary depending on skin type.^2^ The disease is mainly immune mediated, and depends on genetic susceptibility and environmental factors such as UV exposure.^3^ Treatment of CPD is often recalcitrant and challenging. Topical steroids, calcineurin inhibitors, systemic immunosuppressive medications, hydroxychloroquine or phototherapy (‘hardening’) have exhibited varying success.^4^ Janus kinase 1 (JAK1) inhibition using upadacitinib was successfully deployed in a single patient with CAD.^5^ To our knowledge, we report the first case series of CPD successfully treated with upadacitinib, a selective oral JAK1 inhibitor. Patients showed a marked improvement with sustainably controlled disease, reduced sensitivity, in particular reduced burning and itching, and an increased quality of life.

## Case report

### Patient 1

**Figure 1:**
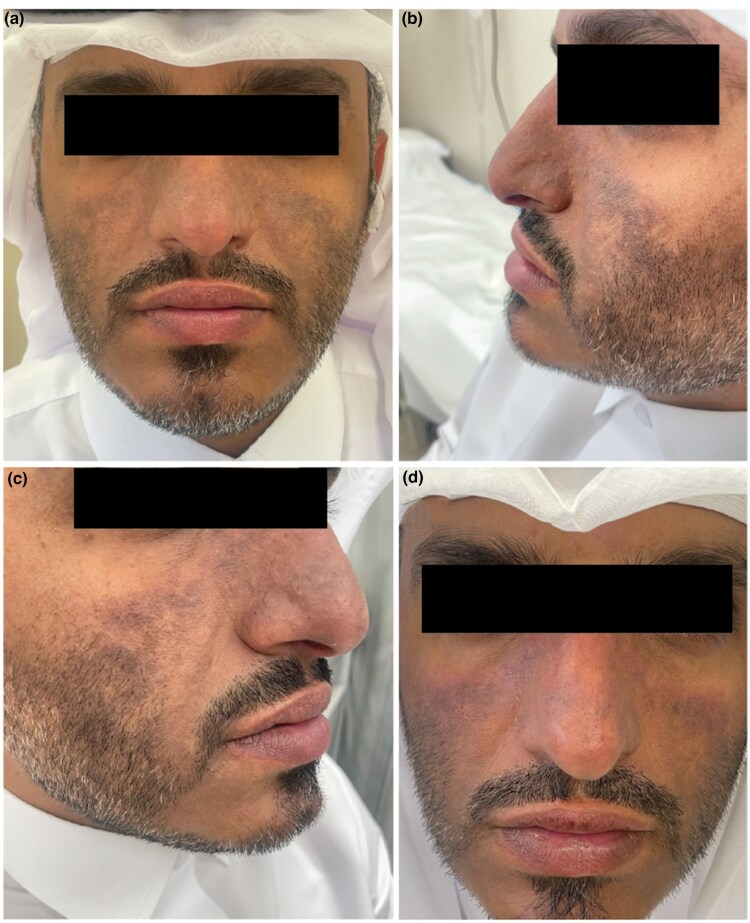
(a–c) A 35-year-old Qatari man with chronic photodermatitis showing prominent centro facial hyperpigmentation symmetrical. (d) Appearance at week 24 after upadacitinib initiation.

A 35-year-old Qatari man with Kartagener syndrome presented with reoccurring facial erythematous, oedematous patches and chronic lichenified hyperpigmentation on his nose and cheeks (Figure 1). The initial symptoms appeared subsequent to a nasal surgery in 2013, he developed centro ­facial erythema. The patient reported severe itch [numerical rating scale (NRS) NRS-11: 9/10] and burning pain (NRS-11: 7/10) (Table 1). The patient’s medical history was positive for asthma and atopic dermatitis (AD). An external histopathological evaluation supported the clinical diagnosis of CPD, revealing a perivascular and periadnexal lymphocytic infiltrate (data not shown). Patch test was unremarkable and antinuclear antibody (ANA) screening was negative. The patient received multiple topical treatments including mometasone, tacrolimus and pimecrolimus without improvement. He was started on oral upadacitinib 15 mg twice daily. After 2 weeks, the patient reported marked improvement of pain and itch, reduced sun sensitivity and erythema, and absent swelling. During follow-up examinations (2.5 and 6 months), the patient presented without signs or symptoms of CPD; however, the patient gained 4 kg of body weight within 2 months of treatment while on a controlled diet and no history of any medical illness, which could be a side effect of the medication.

**Table 1:** Disease severity scoring by case

	Prior to therapy initiation	Week 2	Week 10
Burning sensation			
Patient 1	7/10	2/10	0/10
Patient 2	9/10	0/10	0/10
Patient 3	10/10	0/10	0/10
Itch sensation			
Patient 1	9/10	0/10	0/10
Patient 2	8/10	1/10	0/10
Patient 3	10/10	0/10	0/10

### Patient 2

**Figure 2:**
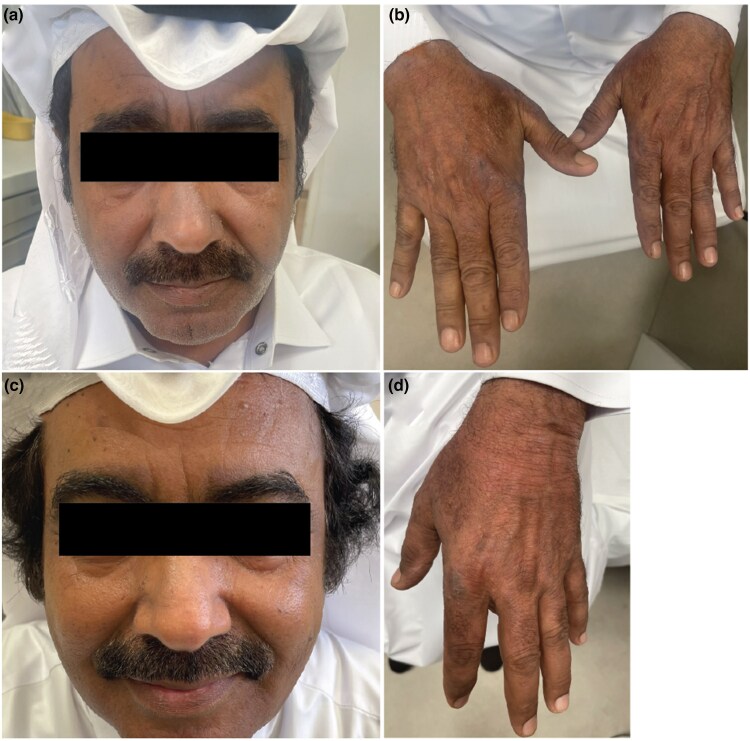
(a, b) A 56-year-old Qatari man with chronic photodermatitis showing unsharply demarcated patches of erythema and hyperpigmentation on the nose and cheek, with scales. (c, d) Appearance at week 20 after upadacitinib initiation.

**Figure 3:**
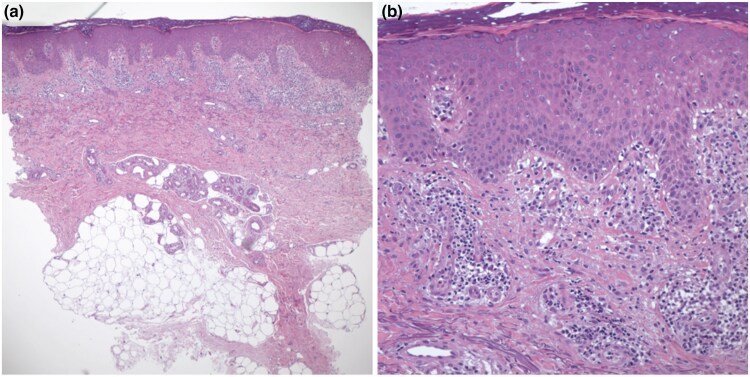
Microscopic examination of lesional skin sections from patient 2. (a, b) Haematoxylin and eosin staining reveals hyperkeratosis and parakeratosis, irregular acanthosis of the epidermis and perivascular inflammation in the upper dermis. (b) Inflammatory infiltrate is composed of lymphocytes, neutrophils and occasional eosinophils. Deep dermis and subcutaneous tissue and adnexal structures are unremarkable (a, ×100; b, ×400).

A 56-year-old Qatari man presented with erythematous and hyperpigmented skin lesions on the face and dorsum of both hands of 1 years’ duration induced by sun exposure. He complained of severe burning pain (NRS-11: 9–10/10) and itch (NRS-11: 8/10) (Table 1). Examination revealed sharply-to-diffusely demarcated patches of erythema and hyperpigmentation on the nose, cheeks and forehead, with scales, and scattered, erythematous and hyperpigmented papules with nodules on the dorsum of both hands (Figure 2). A lesional skin biopsy supported the CPD diagnosis (Figure 3). ANA screening was negative. Topical glucocorticosteroids and systemic antihistamines were tried without benefit. The patient was started on ciclosporin 200 mg twice daily with minimal improvement. Tapering ciclosporin to 100 mg twice daily, the patient developed neurological symptoms. Consequently, oral ciclosporin was stopped, and the patient was started on hydroxychloroquine 200 mg twice daily with topical tacrolimus and pimecrolimus with sunscreen. After 1 month, the patient presented with erythematous skin lesion on the abdomen indicative of a drug reaction; thus, hydroxychloroquine was discontinued. A photosensitivity test resulted in the formation of severe erythema and swelling to a minimum dose of 200 mJ UVA/B within 24 h, with an itch score of 10 /10. Patch test was unremarkable. Due to sun sensitivity and severe burning pain, the patient was started on upadacitinib 15 mg twice daily and topical tacrolimus. Under upadacitinib 15 mg twice daily, the patient showed marked improvement within 2 weeks, with a notable reduction in erythema and swelling and an impressive reduction in itching and burning sensation. No side effects were reported for 8 months.

### Patient 3

A 33-year-old Qatari man presented with superficially scaling erythematous, oedematous skin patches in sun-­exposed areas of the cheeks, nose and forehead starting 6 years previously. The chief complaint was a severe burning pain (NRS-11: 9–10/10) and itch (NRS-11: 9–10/10) on the face and hands (Table 1). The patient’s medical history was positive for atopy. Examination showed diffusely demarcated hyperpigmented facial patches, mainly on both temporal sides and cheeks, with lichenification and scales. The patient had negative autoimmune screening including ANA. The patient had a patch test performed externally with negative results, and external histopathological findings supported the diagnosis of CPD. The patient had been previously treated with topical and systemic glucocorticosteroids (hydrocortisone, betamethasone) and topical pimecrolimus and tacrolimus, all of which worsened his condition. Systemic glucocorticosteroids were associated with common side effects including hypertension and rebound phenomenon after tapering. He was started on systemic dupilumab 300 mg every 4 weeks, but his facial and hand lesions worsened, thus injections were discontinued. A photosensitivity test resulted in the formation of erythema and itchiness within 24 h. Next, the patient was started on upadacitinib 15 mg twice daily, resulting in a marked improvement within 2 weeks, with reduction of swelling and erythema, softening skin texture and, in particular, strongly ameliorated burning pain and itch, which together resulted in improved quality of life.

## Discussion

To our knowledge, we report for the first time a case series of three severe cases of CPD successfully treated with upadacitinib. Targeting the JAK1 signalling pathway with upadacitinib demonstrated a rapid, sustainable efficacy, an excellent safety profile and very good tolerability.

Despite its high prevalence and disease burden there is no consensus for an efficacious, safe and well-tolerated treatment for CPD.^3^ Current treatment can be challenging and may lead to considerable adverse events. First-line treatment of CPD is avoidance of triggers like sun exposure by sunscreen with topical steroids, calcineurin inhibitors or photo(chemo)therapy (UVA/UVB light).^4^ Systemic therapies indicated for severe cases include immunosuppressive medications like oral prednisolone, ciclosporin, azathioprine, methotrexate or hydroxychloroquine, all with variable degrees of success and potentially severe side effects.^4^

Recently, the use of JAK inhibitors has gained increasing attention as an anti-inflammatory, immunomodulating therapy for inflammatory or autoimmune skin diseases such as AD or alopecia areata, for example.^6^ In AD, JAK inhibitors appear to restore the cytokine profile to a more normal state, improving inflammation, barrier function, itch and pain, pathophysiological mechanisms also found in CPD.^7,8^

CPD is an inflammatory skin disease with involvement of cytokines, of which some such as interleukin-31, utilize JAK/signal transducer and activator of transcription signalling.^3,7,8^ Therefore, one may hypothesize that JAK inhibitors are beneficial for the treatment of CPD, ameliorating not only inflammation, but also pain and itch.

Upadacitinib is a selective JAK1 inhibitor, only approved in dermatology for AD, blocking signaling of T-helper 1 (T_H_1)- and T_H_2-associated cytokines.^7^ We decided on initiating a selective JAK1 biologic rather than a pan-JAK inhibitor to avoid reported adverse effects of JAK2 and JAK3 on haematopoiesis, thus reducing the side effect profile for the patients.^9^ CPD is a heterogenous disease with a poorly understood subtype cytokine profile. However, the rapid and sustainable efficacy of upadacitinib as monotherapy in all three patients indicates a central role of JAK1-associated cytokines in CPD.

Taken together, upadacitinib and other JAK inhibitors may be an efficacious, safe and tolerable treatment for CPD. Further studies such as placebo-controlled, randomized clinical trials are required to confirm the benefit of upadacitinib as a systemic treatment for CPD and other photodermatoses.

## Data Availability

All data are incorporated in the article.
